# Evaluating the effects of Esmolol on cardiac function in patients with Septic cardiomyopathy by Speck-tracking echocardiography—a randomized controlled trial

**DOI:** 10.1186/s12871-023-01983-8

**Published:** 2023-02-10

**Authors:** Junyi Wang, Xinjing Gao, Zhengzhong He, Jinxiang Wang, Guowu Xu, Tong Li

**Affiliations:** 1 The Third Central Clinical College of Tianjin Medical University, Tianjin, 300170 China; 2 The Third Central Hospital of Tianjin, 83 Jintang Road, Hedong District, Tianjin, 300170 China; 3 Tianjin Key Laboratory of Extracorporeal Life Support for Critical Diseases, Artificial Cell Engineering Technology Research Center, Tianjin, China; 4 Tianjin Institute of Hepatobiliary Disease, Tianjin, China; 5 Wenzhou Safety (Emergency) Institute of Tianjin University, Wenzhou 325026 Zhejiang, People’s Republic of China; 6 Department of Emergency Medicine, Tianjin Medical University General Hospital, Tianjin, 300052 People’s Republic of China

**Keywords:** Sepsis, Septic Shock, Sepsis Induced Cardiomyopathy, Speckle-tracking Echocardiography

## Abstract

**Background:**

Esmolol as one treatment of sepsis induced cardiomyopathy (SIC) is still controversial. The objective of this study is to evaluate cardiac function after reducing heart rate by Esmolol in patients with SIC using speck-tracking echocardiography.

**Methods:**

This study was a single-center, prospective, and randomized controlled study. A total of 100 SIC patients with a heart rate more than 100/min, admitted to the Intensive Care Department of Tianjin Third Central Hospital from March 1, 2020 to September 30, 2021, were selected as the research subjects. They were randomly divided into the Esmolol group (Group E) and the conventional treatment group (Group C), each with 50 cases. The target heart rate of patients in Group E was controlled between 80/min and 100/min. Speck-tracking echocardiography (STE) and pulse indicating continuous cardiac output monitoring (PICCO) were performed in both groups at 1 h, 24 h, 48 h, 72 h, 96 h and 7 d after admission, with data concerning left ventricular global longitudinal strain (GLS), left ventricular ejection fraction (LVEF) and global ejection fraction (GEF), left ventricular systolic force index (dP/dtmx) were obtained, respectively. Hemodynamics and other safety indicators were monitored throughout the whole process. These subjects were followed up to 90 d, with their mortality recorded at Day 28 and Day 90, respectively. Statistical analyses were performed using SPSS version 21.

**Results:**

With 24 h of Esmolol, all patients in Group E achieved the target heart rate, and there was no deterioration of GLS, or adverse events. However, compared with those in Group C, their GLS, GEF and dP/dtmx were increased, and the difference was statistically significant (*P* > 0.05). Compared with patients in Group C, those in Group E had lower short-term mortality, and logistic regression analysis also suggested that Esmolol improved patient outcomes.

**Conclusion:**

In SIC patients, the application of Esmolol to lower heart rate decreased their short-term mortality while not making any impairment on the myocardial contractility.

**Trial registration:**

Chinese Clinical Trial Registry, ChiCTR2100047513. Registered June 20, 2021- Retrospectively registered, http://www.chictr.org.cn/index.aspx. The study protocol followed the CONSORT guidelines. The study protocol was performed in the relevant guidelines.

**Supplementary Information:**

The online version contains supplementary material available at 10.1186/s12871-023-01983-8.

## Background

Sepsis is considered the main cause of death in critically ill patients worldwide [1, 2]. Heart is susceptible to Sepsis and is clinically manifested as Sepsis Induced Cardiomyopathy (SIC), which has an incidence of 50% in Sepsis patients and even up to 70% in Septic Shock patients [3, 4]. SIC is also one of the causes of increased mortality in patients with Sepsis or Septic Shock [5–7]. In the case of Sepsis, the host fights against infection by activating the hemodynamic, metabolic, and immune processes. During the progress, it will partly activate the sympathetic nervous system and promote the release of catecholamine hormones [8]. Recommended as the first-line vasoactive agent in the treatment of Sepsis by the guidelines, norepinephrine is extensively adopted in clinical practice. Excessive stress caused by the release of endogenous catecholamines and the application of exogenous catecholamines acts directly on the myocardium of patients with Sepsis, resulting in increased myocardial contractility and tachyarrhythmia [9], an independent risk factor for death in patients with severe Sepsis and Septic Shock [10, 11].

Tachyarrhythmia can impair ventricular filling and increase myocardial oxygen consumption, making the use of β -blockers for correcting tachyarrhythmia a treatment option for SIC. Currently, the use of β-blockers in the treatment of SIC patients is still controversial [7, 12]. Previous studies have shown that Esmolol can improve the 28-day mortality in patients with Sepsis, and is safe and effective in reducing heart rate, exerting no significant adverse effects on tissue perfusion [13–17]. However, when Sepsis occurs, especially after Septic Shock, the hemodynamics are in an unstable state, while β-blockers act directly on myocardium, causing a decrease in myocardial contractility, and further aggravating hemodynamic instability [18]. To this end, it is urgent to find an effective and feasible method to evaluate the changes of myocardial contractility in the treatment of SIC with β -blockers and monitor hemodynamics closely.

The diagnosis of SIC is still limited to the left ventricular ejection fraction (LVEF) obtained by transthoracic echocardiography (TTE) [19, 20], which is affected by various factors, making its clinical application widely doubted. Speckle-tracking echocardiography (STE) is a new echocardiographic method that can better evaluate cardiac function. The global longitudinal strain (GLS) is the most commonly used strain measurement method and represents the ratio of the maximum longitudinal length change during myocardial contraction to the original length during diastole. Less affected by the pre- and post-load heart state, it can better evaluate myocardial function and is considered to improve the accuracy of SIC diagnosis [21, 22].

The hypothesis was proposed that Esmolol can effectively reduce heart rate in SIC patients without damaging myocardial contractility and worsening hemodynamics. The purpose of this study was to evaluate myocardial contractility with STE after the heart rate was reduced to the target value with the application of Esmolol, with its effect on 28-day and 90-day mortality recorded.

## Method

Patients admitted to the Intensive Care Department of Tianjin Third Central Hospital (Grade III, Class A) with 30 comprehensive ICU beds from March 1, 2020 to September 30, 2021 were selected as the study subjects, and the study was approved by the Ethics Committee of Tianjin Third Central Hospital. All subjects were followed up to 90 days after discharge, with death as an end point.

### Inclusion criteria

Patients diagnosed with Sepsis/Septic Shock according to Sepsis-3 [23] within 1 h after admission, and still presenting tachyarrhythmia (HR ≥ 100 bpm) after completing 1-h bundle and 24 h of standardized treatment according to the Surviving Sepsis Campaign-2018 [24]; echocardiography at 24 h suggesting LVEF ≤ 45% or GLS ≥ -19% [25, 26]; and age ≥ 18 years old (eTable 1).

### Exclusion criteria

Transferred patients diagnosed with Sepsis before admission and treated in another hospital for more than 48 h; history of atrial fibrillation, long-term use of β-blockers; history of chronic heart failure (NYHA ≥ grade III); history of moderate/severe valvular heart disease; rapid arrhythmias such as new-onset atrial fibrillation/atrial flutter; new-onset cardiogenic shock (LVEF ≤ 25%); poor echocardiography image quality; refusing to sign informed consent; age < 18 years old; pregnant and lactating women; or patients with severe malignant tumors.

### Study design

This study was a single-center, prospective, and randomized controlled study. Patients were randomly divided into the Esmolol group (Group E) and the conventional treatment group (Group C) using the random number table method, each with 50 cases. Both groups were treated under the guidance of Surviving Sepsis Campaign – 2018, and achieved the goal of having a CVP greater than 10 mmHg and a negative volume reactivity within 24 h. Only norepinephrine was used to maintain blood pressure throughout the treatment, and other inotropes and vasopressors were not added.

### Sample size calculation

Compared with Group C, GLS in Group E improved after 24 h of Esmolol application in the previous preliminary test [(-11.54 ± 4.67) VS (-8.72 ± 4.23)], and hemodynamics did not deteriorate. The sample size was calculated using PASS 14.0 software, and the parameters were set as: β ≤ 0.1; power (1-β) = 90%; and significance level bilateral α = 0.05. The calculated sample size was 46 cases in each group, and 50 were included in each group according to the shedding rate of 20%.

### Esmolol

Group E was given Esmolol at 24 h (specification: 10 ml/0.1 g; manufacturer: Qilu Pharmaceutical Co., LTD.) to maintain a heart rate between 80/min and 100/min. The pharmacological characteristics of Esmolol show that bolus doses of 100 to 200 mg are effective in attenuating the adrenergic responses, followed by continuous intravenous infusion at dose of less than 300 ug/kg/min to control the heart rate[27]. The plasma concentration of Esmolol is proportional to the dose, and the risk of hypotension increases with the higher dose of Esmolol[28]. According to the drug label of Esmolol, a low dose of Esmolol was used as the starting dose for titration, with a loading dose of 0.5 mg/kg followed by continue intravenous pumping at 0.05 mg/kg/min for maintenance. If the effect is not favorable after 4 min, give the loading dose again and increase the maintenance dose by 0.05 mg/kg/min. The maximum maintenance dose can be increased to 0.2 mg/kg/min. For patient safety, vital signs and hemodynamic changes are closely monitored during Esmolol titration, and if endpoint events occurred, Esmolol infusion was promptly reduced or discontinued (Additional file 1, eFigure2). We continued infusing Esmolol to maintain the predefined heart rate threshold until either ICU discharge or aggravation of septic shock that is not suitable for continued use of Esmolol reported by clinicians. Hemodynamic variables were determined at baseline and after 24 h of Esmolol infusion.

### STE and PICCO

All patients were examined for STE and PICCO within 1 h, 24 h, 48 h, 72 h, 96 h and 7d, respectively after admission. STE was finished by an experienced ultrasound physician using GE Vivid Q color Doppler bedside ultrasound with M5s probe, and the frequency was 1.7 ~ 3.4 MHz. He only know the study protocol, but blind to the grouping protocol. The PICCO examination was performed by scientific research physicians using PICCO-3.

### Outcome

This study evaluated changes in myocardial contractility after Esmolol lowered heart rate mainly using GLS, and selected the values of GLS at 48 h after admission, when, the heart rate reached target heart rate after using Esmolol, becoming the prime outcome. Daily dosage of norepinephrine, ratio of reaching target heart rate, values of GEF and dP/dtmax, length of ICU and in-hospital, duration of mechanical ventilation and follow-up records of 28d and 90d were considered secondary outcomes.

### Security monitoring

#### Hemodynamics

All subjects were indwelled with internal jugular vein catheter and femoral artery catheter for PICCO monitoring after hospitalization. Baseline hemodynamic data, including heart rate (HR), systolic pressure (SBp), diastolic pressure (DBp), mean arterial pressure, (MBp), cardiac Index (CI), central venous pressure (CVP), left ventricular contractility index (dP/dtmax), global ejection fraction (GEF), global end-diastolic volume Index (GEDVI) and extravascular lung water index (ELWI), were recorded at 0 h, 24 h, 48 h, 72 h, 96 h and 7d.

#### Clinical data

Baseline laboratory indicators, including lactic acid (Lac), central venous oxygen saturation (ScVO2), central venous-arterial carbon dioxide difference (P_v-a_CO2), amino-terminal brain natriuretic peptide (NT-proBNP), creatine kinase (CK), creatine kinase isoenzyme (CK-MB), troponin I (TNI) and oxygen delivery (DO2), were collected at 0 h, 24 h, 48 h,72 h, 96 h and 7d, and calculating the APACHE II and SOFA score respectively.

#### Statistical analysis

Statistical analyses were performed using SPSS version 21. Firstly, the normal distributed data were described by mean ± standard deviation, and the median and quartile range were used for descriptive statistics for non-normally distributed data; Secondly, the difference test of grouped variables was carried out based on the research purpose. Normally distributed data were compared using independent student's T-test, and nonparametric U Mann–Whitney test was used for non-normally distributed data. Chi-square test or Fisher's test was used to compare categorical variables. Among the test results, *P* value less than 0.05 was considered to have significant statistical difference. Besides, the 28-day and 90-day survival in Group E and Group C was evaluated using the Kaplan–Meier method; Finally, logistic regression model was established for analyzing the effect of Esmolol on the prognosis of patients.

## Results:

**Fig. 1:**
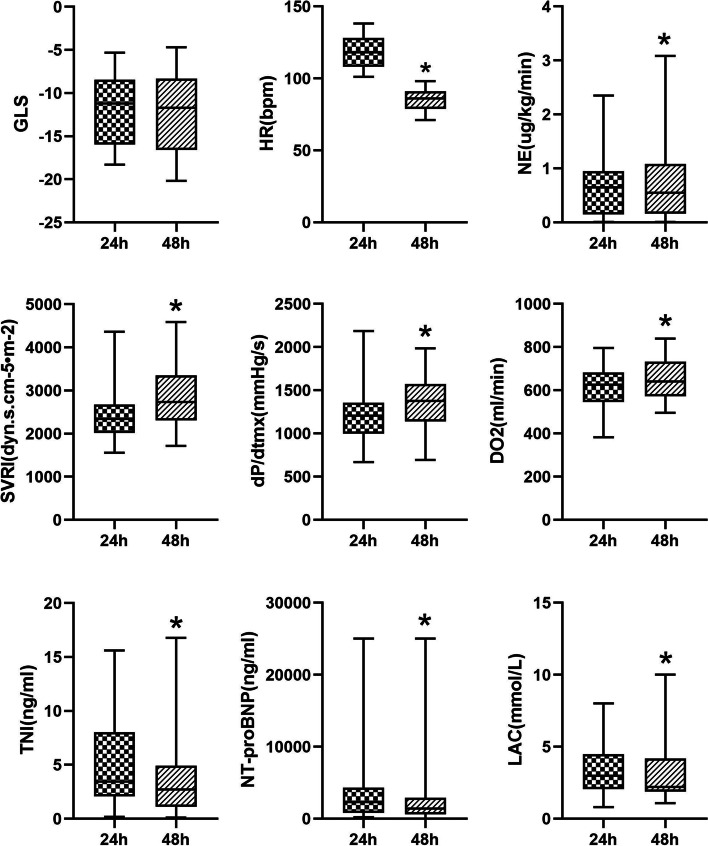
Comparison between 24 and 48 h in ICU in Group E. *:*P* < 0.05

**Fig. 2:**
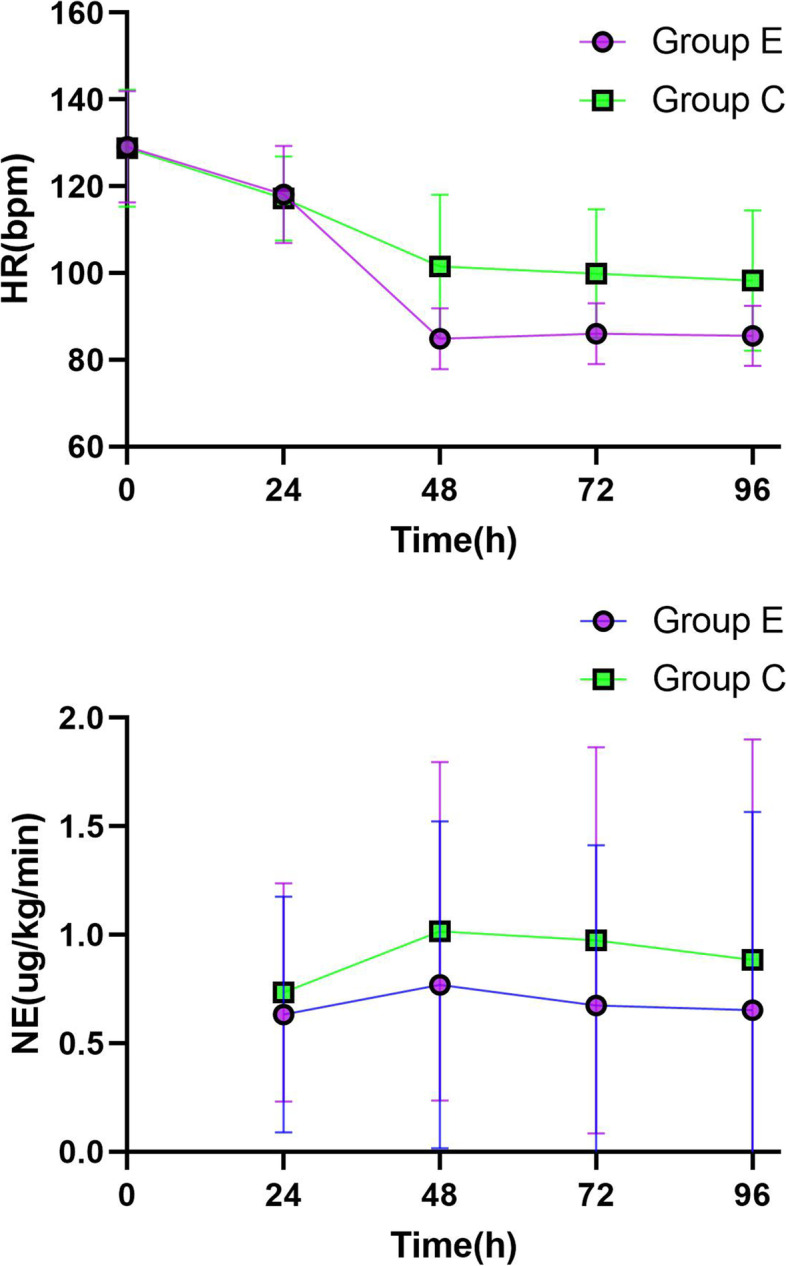
Changes of HR and NE dosage over time in the two groups. The HR and norepinephrine dosage of the two groups showed a downward trend with time, especially in group E

**Fig. 3:**
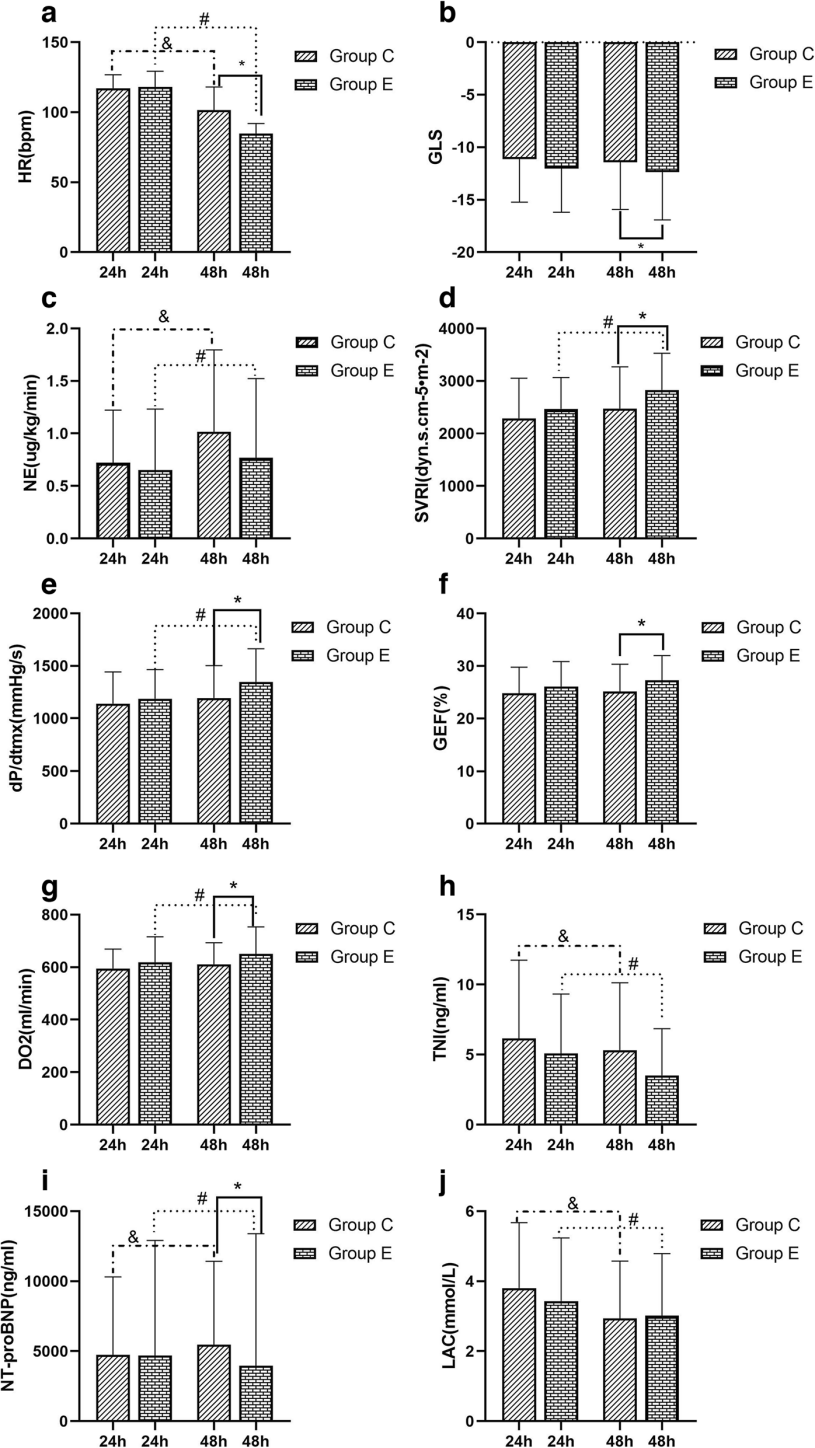
Comparison between Group E and Group C at 24 h and 48 h in ICU. *: *P* < 0.05 when comparison of indicators between Group E and Group C at 48 h; *#*: *P* < 0.05 in the comparison of indicators in Group E at 24 h and 48 h; *&*: *P* < 0.05 in the comparison of indicators in Group R at 24 h and 48 h

**Fig. 4:**
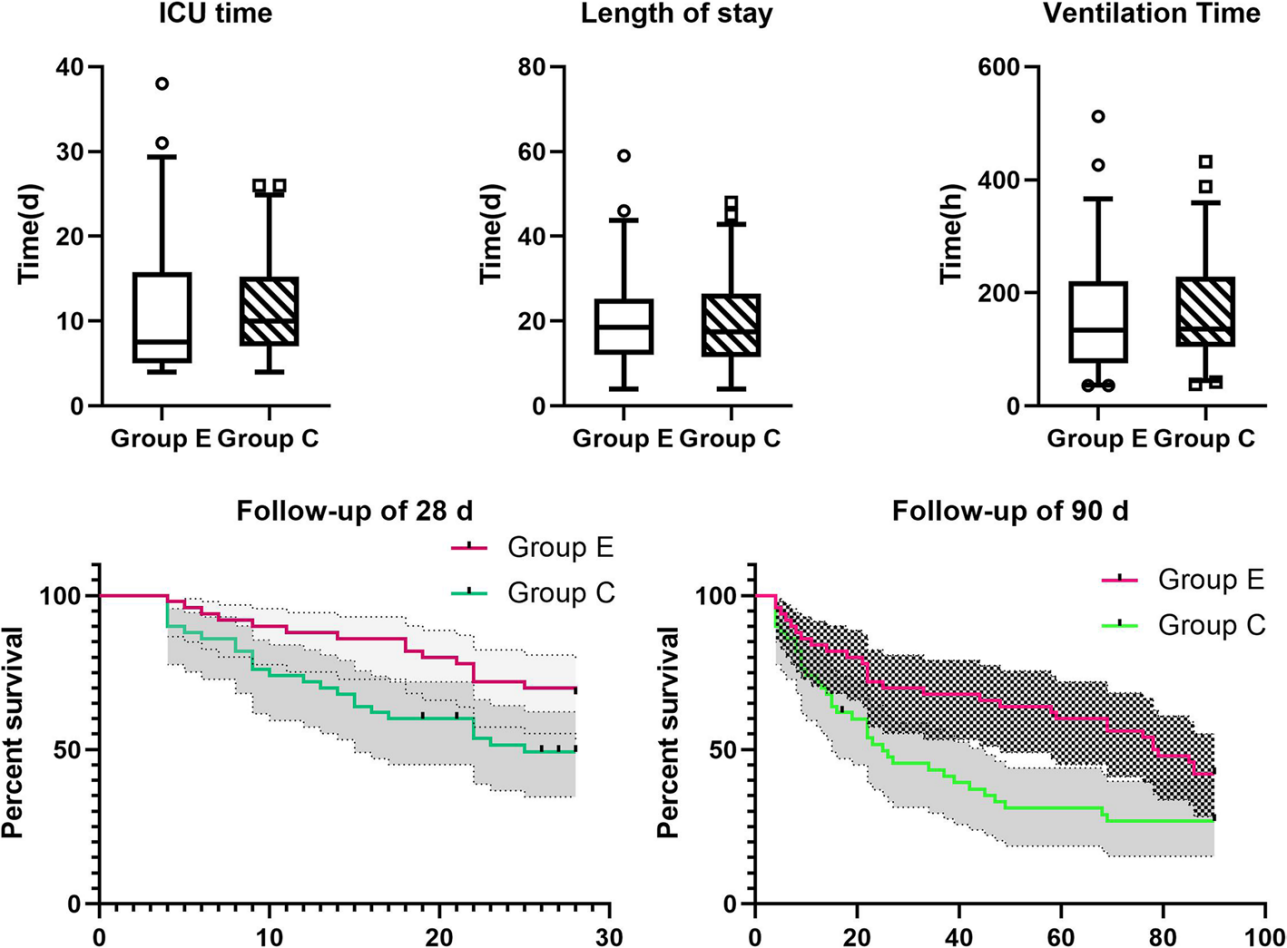
Outcomes between Group E and Group C. The median duration of follow-up was 28 days and 90 days in both study groups. Vertical ticks on the curves indicate censoring due to loss to follow-up after hospital discharge. d: day; h: hours

1. A total of 324 patients with Sepsis/Septic Shock were hereby screened, among which, 100 with SIC were finally included, and randomly divided into Group E (50 cases) and Group C (50 cases according to the inclusion and exclusion criteria). Baseline data, including age, sex, BMI, SOFA score, APACHE II score, HR, MBp, LAC, 24-h fluid balance, Sepsis etiology and previous chronic diseases, were similar between the two groups, and the differences were not statistically significant (Table 1);
2. All patients in Group E reached the target heart rate [(118.1 ± 13.19) VS (84.86 ± 7.03), *P* < 0.05] 24 h after Esmolol application, and GLS was not deteriorate [(-12.02 ± 4.16) VS (-12.36 ± 4.55), *P* > 0.05] and no adverse events occurred. At the same time, a reduce in LAC was observed after Esmolol application [(3.43 ± 1.80) VS (3.01 ± 1.77),*p* < 0.05], [5.12(3.05, 10.28) VS 3.45(2.01, 7.89), Z = -4.112, 2321.52(835.52, 4304.94) VS 1412.66(620.62, 2924.58), Z = -4.063, *p* < 0.05] for TNI and NT-proBNP. In addition, the study also found an increase in SVRI, SV, ScvO2 and DO2, also an increase in the dose of norepinephrine [(2828.38 ± 698.46) VS (2469.9 ± 596.35), (46.79 ± 15.73) VS (63.01 ± 19.20), (67.86 ± 7.45) VS (70.56 ± 4.77), (619.06 ± 96.45) VS (650.54 ± 103.37), (0.63 ± 0.54) VS (0.77 ± 0.75), P < 0.05] (Table 2, Fig. 1).
3. After Esmolol was applied for 3 consecutive days, the heart rate of patients in Group E was maintained at the target heart rate, while the norepinephrine dosage presented a decreasing trend. The heart rate and norepinephrine dosage of those in Group C also decreased over time, but failing to reach the target value compared to those in Group E (Fig. 2).
4. At 48 h in ICU, parameters including GLS, GEF and dP/dtmx reflecting myocardial contraction for patients in Group E were better than those in Group C [(-12.36 ± 4.56) VS (-10.49 ± 4.51), (27.26 ± 4.82) VS (24.78 ± 5.02), (1347.1 ± 315.2) VS (1140.9 ± 301.0), *p* < 0.05]. At the same time, NT-proBNP in Group E was found lower than that in Group C [1412(620, 2924) VS 2772(861, 6995), Z = -2.058, *p* < 0.05], and even the norepinephrine dosage of the two groups of patients in a similar situation, SVRI was higher in Group E patients than those in Group C [(2828.4 ± 698.5) VS (2474.0 ± 793.3), *p* < 0.05]. Moreover, DO2 in Group E was improved [(650.54 ± 103.38) VS (611.22 ± 82.01), *p* < 0.05] (Table 3, Fig. 3). At 24 h in ICU, there was no statistical difference in parameters between the two groups (*p* > 0.05). Compared with 24 h in ICU, heart rate, TNI and LAC of patients in Group C decreased at 48 h in ICU, while the dosage of norepinephrine and the level of NT-proBNP increased, showing statistically significant differences (Fig. 3). At 72 h in ICU, it was also found that parameters including GLS, GEF and dP/dtmx in group E were better than those in group C, while there was no difference between the two groups at 96 h and day 7 in ICU (Additional file 1, eTable 1).
5. There was no statistically significant difference between the two groups in terms of duration of mechanical ventilation, length of ICU and in-hospital (*p* > 0.05) (Table 4). However, using Kaplan–Meier to analyze the 28-day and 90-day mortality, significant statistical differences were observed between the two groups [16(32%) VS 25(50%), 29(58%) VS 36(72%), (*p* < 0.05)] (Fig. 4).
6. Patients were divided into Group Death and Group Survival according to the outcomes. Using logistic regression model to analyze the effect of Esmolol on patient outcomes, it was found that after adjusting for SOFA score and DO2 factors, patients treated with Esmolol had a reduced risk of death at Day 28 (OR = 3.127, 95% CI:1.151–8.494) (Table 5).

**Table 1:** Demographics for each cohort

Variables	Full cohort (*n* = 100)	Group E (*n* = 50)	Group C (*n* = 50)
Age, Median (IQR), y	67.5(57.25–77)	69 (58–77.25)	67.5 (56.75–77)
Men (%)	57(*n* = 57)	58 (*n* = 29)	56 (*n* = 28)
Body mass index, Median (IQR)	25.15 (21.78–28.38)	25.95 (24.16–25.95)	22.78 (21.64–24.16)
SOFA score, Median (IQR)	11(8–13)	11(8.75–13.25)	10(8–13.25)
Apache II score, Median (IQR)	28(24.25–33)	27.5(21.75–33)	29(26–32.25)
HR, Median (IQR)	118(108.25–126)	118(108–128)	118(108.75–122.5)
MBP, Median (IQR)	83(73.25–92.75)	81(71.5–94)	83.5(75–91.5)
LAC, Median (IQR)	3.11(2.15–4.81)	2.99(2.06–4.49)	3.14(2.21–5.12)
Fluid input balance in 24 h (ml), Median (IQR)	2250 (1470–3070)	2027 (1542–2891.25)	2367 (1614–3217.75)
Sepsis source (%)			
Pneumonia	43(*n* = 43)	38(*n* = 19)	48(*n* = 24)
Hepatapostema	7(*n* = 7)	8(*n* = 4)	6(*n* = 3)
Cholangitis	12(*n* = 12)	12(*n* = 6)	12(*n* = 6)
Peritonitis	20(*n* = 20)	18(*n* = 9)	22(*n* = 11)
Nephropyelitis	4(*n* = 4)	2(*n* = 1)	6(*n* = 3)
Other	14(*n* = 14)	22(*n* = 11)	6(*n* = 3)
Preexisting conditions (%)			
Diabetes	53(*n* = 53)	50(*n* = 25)	56(*n* = 28)
Coronary heart disease	60(*n* = 60)	64(*n* = 32)	56(*n* = 28)
Previous myocardial infarction	28(*n* = 28)	36(*n* = 18)	20(*n* = 10)
Hypertension	44(*n* = 44)	46(*n* = 23)	42(*n* = 21)
Cerebrovascular accident,	27(*n* = 27)	24(*n* = 12)	30(*n* = 15)
Uremia	6(*n* = 6)	2(*n* = 1)	10(*n* = 5)

Continuous variables expressed as median (Q1, Q3), Nominal variables expressed as number (%)

*SOFA* Sequential organ failure assessment, *Apache II* Acute physiology and chronic health, *HR* Heart rate, *MBP* Mean blood pressure, *LAC* Lactic acid

**Table 2:** Variable comparison between 24 and 48 h in ICU in Group E

Variables	ICU 24 h	ICU 48 h	P
NE(μg·kg^−1^·min^−1^)	0.63 ± 0.54	0.77 ± 0.75	0.002*
HR(bpm)	118.1 ± 13.19	84.86 ± 7.03	0.000*
MBP(mmHg)	83.20 ± 13.19	81.12 ± 12.54	0.174
GLS	-12.02 ± 4.16	-12.36 ± 4.55	0.160
E/e’	14.39 ± 5.37	16.97 ± 20.76	0.363
LVEF(%)	46.20 ± 11.04	47.46 ± 10.32	0.154
SV(ml)	46.79 ± 15.73	63.01 ± 19.20	0.000*
CI(L/min/m^2^)	3.38 ± 0.92	3.34 ± 0.86	0.509
SVRI(dyn.s.cm^−5^·m^−2^)	2469.9 ± 596.35	2828.38 ± 698.46	0.000*
GEDI(ml/m^2^)	757.72 ± 58.85	749.98 ± 55.85	0.472
GEF(%)	26.04 ± 4.81	27.26 ± 4.72	0.073
dP/dtmx(mmHg/s)	1187.74 ± 276.30	1347.08 ± 315.17	0.000*
CVP(mmHg)	11.06 ± 2.66	11.56 ± 3.42	0.127
Lac(mmol/L)	3.43 ± 1.80	3.01 ± 1.77	0.004*
VO2(ml/min)	263.40 ± 78.61	256.30 ± 85.28	0.272
DO2(ml/min)	619.06 ± 96.45	650.54 ± 103.37	0.008*
P_(v-a)_CO_2_(mmHg)	5.46 ± 2.50	5.15 ± 2.14	0.171
ScvO2(%)	67.86 ± 7.45	70.56 ± 4.77	0.008*
PO2/FiO2(mmHg)	236.74 ± 89.51	266.92 ± 98.09	0.000*
TNI(ng/ml)	5.12(3.05, 10.28)	3.45(2.01, 7.89)	0.000*
NT-proBNP(ng/ml)	2321.52(835.52, 4304.94)	1412.66(620.62, 2924.58)	0.000*

Continuous variables expressed as means ± SD or median (Q1, Q3)

*NE* Norepinephrine, *HR* Heart rate, *bpm* Beats per minute, *MBP* Mean blood pressure, *GLS* Left ventricular global longitudinal systolic strain, *LVEF* Left ventricular ejection fraction, *SV* Stroke volume, *CI* Cardiac index, *SVRI* Systemic vascular resistance index, *GEDI* Global end diastolic volume index, *GEF* Global ejection fraction, *dP/dtmx* Left ventricular contractility index, *CVP* Central venous pressure, *VO2* Oxygen consumption, *DO2* Oxygen delivery, *P*_*(v-a)*_*CO*_*2*_ Central venous-arterial carbon dioxide partial pressure difference, *ScvO2* Central venous oxygen saturation, *TNI* Troponin I, *NT-proBNP* N-terminal pro-brain natriuretic peptide

^*^^:^ *p* < 0.05

**Table 3:** Comparison between Group E and Group C at 24 h and 48 h in ICU

Variables	ICU24h		P	ICU48h		P
	**Group E**	**Group C**		**Group E**	**Group C**	
NE (μg•kg-1•min-1)	0.63 ± 0.54	0.72 ± 0.50	0.387	0.77 ± 0.75	1.02 ± 0.78	0.111
HR(bpm)	118.1 ± 11.1	117.2 ± 9.7	0.667	84.9 ± 7.0	101.5 ± 16.5	0.000*
MBP(mmHg)	83.2 ± 12.2	84.1 ± 10.8	0.704	81.1 ± 12.5	83.2 ± 8.41	0.333
GLS	-12.02 ± 4.16	-11.1 ± 4.1	0.826	-12.36 ± 4.56	-10.49 ± 4.51	0.042*
E/e’	14.39 ± 5.37	14.25 ± 5.69	0.900	14.18 ± 5.87	14.45 ± 5.96	0.825
LVEF(%)	46.2 ± 11.0	48.9 ± 12.0	0.234	48.3 ± 10.8	49.9 ± 11.9	0.470
SV(%)	46.79 ± 15.73	45.25 ± 14.14	0.608	63.01 ± 19.20	49.08 ± 14.94	0.000*
CI(L/min/m2)	3.39 ± 0.92	3.15 ± 1.00	0.224	3.34 ± 0.86	3.07 ± 0.89	0.133
SVRI (dyn.s.cm-5•m-2)	2469.9 ± 596.4	2287.0 ± 763.1	0.185	2828.4 ± 698.5	2474.0 ± 793.3	0.020*
GEDI(ml/m2)	757.7 ± 58.5	775.9 ± 87.6	0.225	750.0 ± 55.9	758.3 ± 79.8	0.549
EWLI(ml/kg)	9.44 ± 3.30	9.26 ± 3.93	0.805	9.28 ± 2.85	9.26 ± 3.93	0.977
GEF(%)	26.04 ± 4.82	24.78 ± 5.02	0.203	27.26 ± 4.82	24.78 ± 5.02	0.013*
dP/dtmx (mmHg/s)	1187.7 ± 376.4	1140.9 ± 301.0	0.419	1347.1 ± 315.2	1194.2 ± 308.5	0.016*
CVP(mmHg)	11.06 ± 2.67	10.88 ± 3.03	0.753	11.56 ± 3.42	10.88 ± 3.03	0.295
Lac(mmol/L)	3.43 ± 1.80	3.80 ± 1.87	0.320	3.01 ± 1.77	2.93 ± 1.64	0.812
VO2(ml/min)	263.40 ± 78.62	268.34 ± 70.21	0.741	256.30 ± 85.28	265.82 ± 69.53	0.542
DO2(ml/min)	619.06 ± 96.45	595.00 ± 74.29	0.165	650.54 ± 103.38	611.22 ± 82.01	0.038*
P(v-a)CO2 (mmHg)	5.46 ± 2.50	6.25 ± 2.50	0.119	5.15 ± 2.15	4.71 ± 2.19	0.313
ScvO2	67.86 ± 7.45	68.02 ± 5.76	0.906	70.56 ± 4.77	67.87 ± 6.16	0.016*
PO2/FiO2(mmHg)	236.74 ± 89.51	244.16 ± 80.78	0.664	266.92 ± 98.09	265.02 ± 96.23	0.992
TNI(ng/ml)	5.12(3.05, 10.28)	4.49(1.51, 10.92)	0.540	3.45(2.01, 7.88)	3.36(1.35, 9.05)	0.926
NT-proBNP(ng/ml)	2321(838, 4304)	2373(766, 7564)	0.664	1412(620, 2924)	2772(861, 6995)	0.040*

Continuous variables expressed as means ± SD or median (Q1, Q3)

*NE* Norepinephrine, *HR* Heart rate, *bpm* Beats per minute, *MBP* Mean blood pressure, *GLS* Left ventricular global longitudinal systolic strain, *LVEF* Left ventricular ejection fraction, *SV* Stroke volume, *CI* Cardiac index, *SVRI* Systemic vascular resistance index, *GEDI* Global end diastolic volume index, *GEF* Global ejection fraction, *dP/dtmx* Left ventricular contractility index, *CVP* Central venous pressure, *VO2* Oxygen consumption, *DO2* Oxygen delivery, *P(v-a)CO2* Central venous-arterial carbon dioxide partial pressure difference, *ScvO2* Central venous oxygen saturation, *TNI* Troponin I, *NT-proBNP* N-terminal pro-brain natriuretic peptide

^*^^:^ *p* < 0.05

**Table 4:** Outcomes between Group E and Group C

Variables	Group E	Group C	P
ICU time (d)	10.96 ± 7.99	11.62 ± 5.99	0.555
Length of stay (d)	20.10 ± 11.84	19.08 ± 10.83	0.608
Ventilation time (h)	156.08 ± 103.14	172.72 ± 90.12	0.331

**Table 5:** Logistic regression analysis of 28-day risk factors for death

	**B**	**SE**	**Wals**	**Sig**	**Exp (B)**	**95% C.I. of EXP(B)**	
						**Lower**	**Upper**
Esmolol	1.140	0.510	5.000	0.025*	3.127	1.151	8.494
SOFA	0.404	0.097	17.279	0.000*	1.497	1.238	1.811
DO_2_	0.009	0.004	6.793	0.009*	1.009	1.002	1.016
Constant	-7.968	1.715	21.584	0.000	0.000		

*SOFA* Sequential organ failure assessment, *DO2* Oxygen delivery

^*^: *p* < 0.05

## Discussion

Given its negative inotropic effect, the application of Esmolol is still widely controversial as one treatment of SIC. This study showed that all SIC patients achieved the target HR after Esmolol, and there was no significant reduction in left ventricular contractility, and the indicators of GEF and dP/dtmx were increased, and the short-term mortality rate was reduced compared with those with uncontrolled HR.

Whether the use of β-blockers in the treatment of SIC worsens hemodynamics remains controversial as well. A large multi-center study showed that early application of β-blockers during acute myocardial infarction reduced the risk of reinfarction and ventricular fibrillation, but increased the incidence of cardiogenic shock, especially at the first day after admission [29]. In addition, a meta-analysis for heart failure after myocardial infarction also suggested that the use of β-blockers within 24 h after myocardial infarction would increase the mortality incidence of patients with cardiogenic shock [18], which might be attributed to the negative inotropic effect of β-blockers. Helen Paur et al. found that the application of β-blockers would aggravate the negative inotropic effect of adrenaline dependence in the case of stress cardiomyopathy [30]. Proceeding from this controversial point, further investigation was conducted on whether the myocardial contractility of SIC patients was impaired after β-blockers lowering the HR.

Although there is no uniform definition of SIC at present, LVEF decline is often considered a diagnostic criterion [20]. Given that GLS is less affected by cardiac preload and afterload, and can detect cardiac function damage earlier than LVEF, it is frequently recommended to replace LVEF as a new method evaluating left ventricular function [31–33]. To this end, GLS was hereby adopted as an indicator to guarantee the evaluation accuracy, and GEF and dP/dtmx were used as well. It can be found from the results of the present study that after Esmolol lowering the HR of patients in Group E, indicators of GLS, GEF and dP/dtmx were not significantly worsened, and SVRI and SV were increased. At the same time, compared with Group C, indicators of GLS, GEF, dP/dtmx, SVRI and NT-proBNP were improved in Group E, and the difference was statistically significant. The results can be explained from the following three points: First, in order to suppress the excessive inflammatory response in Sepsis, the secretion of endogenous catecholamine hormones is increased through the neuroinflammatory reflex in the body [34]. In the meanwhile, the body also mobilizes the adrenergic effect to increase heart function [35] and correct the microcirculation disorder and tissue hypoxia caused by the imbalance of oxygen supply and demand during Sepsis/Septic Shock. Over-stimulation of catecholamines may lead to the collapse of the cardiovascular system, presenting weakened myocardial contractility and tachyarrhythmia that can damage ventricular filling, cause myocardial ischemia, and further damage myocardial contractility. However, Esmolol can improve ventricular filling and myocardial perfusion after lowering HR, thereby avoiding damage to myocardial contractility. A meta-analysis of β-blockers used in patients with severe Sepsis and Septic Shock confirmed their efficacy to improve the prognosis of patients without causing hemodynamic deterioration [36]. Secondly, existing studies have summarized the pathophysiological mechanism of SIC as excessive release of inflammatory factors during Sepsis, which leads to a series of direct injuries, such as cardiovascular dysfunction, imbalance of calcium homeostasis, mitochondrial dysfunction, and down-regulation of β-adrenergic receptor expression, and causes cardiac insufficiency ultimately [19–21]. Takeshi Suzuki et al. found that Esmolol could inhibit the release of pro-inflammatory factors in their study on the regulation of cardiac function in Sepsis mice [37]. Luyao Zhang et al. proposed in the study of Esmolol on mice with severe pancreatitis that continuous infusion of Esmolol could reduce inflammatory response, and protect lung and pancreas [38]. The above studies have confirmed that Esmolol effectively protects the myocardium by inhibiting the release of inflammatory factors. Finally, studies have shown that ventricular-arterial decoupling persists during Septic Shock. Increased static arterial elasticity caused by tachycardia will increase ventricular-arterial decoupling [39, 40]. A. Morelli et al. found in a prospective observational study on the correlation between Esmolol reducing HR and improving arterial elasticity in patients with Septic Shock that Esmolol could reduce static arterial elasticity and restore the ventricular-artery coupling, thereby improving cardiovascular efficacy [41]. Wei Du et al. also claimed that Esmolol may restore vascular waterfall after acute endotoxemia [42]. Antoine Kimmoun et al. proposed that selective β1 blockers can enhance cardiac intrinsic contractile force and vascular responsiveness to catecholamines when added to standard Septic Shock therapy [43]. All these indicate that Esmolol can improve ventricular-artery coupling and vascular tension after stabilizing HR, thereby increasing myocardial contractility.

In the present study, it was also found that the dosage of norepinephrine was very high at baseline in both groups. As is showed in baseline demographic and clinical characteristics, such as SOFA score and APACHE II score, the patients had a higher degree of critical ills, which were associated with the high dose of norepinephrine at baseline. Our hospital is a tertiary A-level hospital, and is also the location of the provincial critical care medicine quality control center where the patients admitted to are in a high degree of critical illness. The patients admitted to the ICU always suffer from various critical diseases and most of them are whose disease had progressed after treatment in other wards of the hospital. Patients were already suffering from severe sepsis or septic shock when transferred to the ICU, which may have contributed to the high dosage of norepinephrine use at baseline. The dosage of norepinephrine in group E increased after Esmolol. However, norepinephrine dose also increased in group C at 48 h, and there was no significant difference compared with group E. In addition, Fig. 2 shows that the dosage of norepinephrine decreased over time in both groups. These results suggest that an increase in norepinephrine dose after Esmolol was associated with a higher degree of critical illness and instability or progression of the disease, rather than the effect of Esmolol. Martin Balik et al. found no increase in the dose of norepinephrine after applying Esmolol in patients with Septic Shock [44]. Fuchs et al. also proposed that the use of vasoactive drugs did not increase in a study of the correlation between the use of Esmolol and 90-day mortality in patients with severe Sepsis and Septic Shock [45]. Bruno Levy et al. found in a study of the hemodynamic effects of early use of Esmolol in patients with highly dynamic Septic Shock that rapid use of Esmolol for controlling HR in the early stages of Septic Shock led to a decrease in cardiac index and an increased risk of hypotension [46]. In order to reduce the influence of Esmolol on hemodynamics, all subjects were hereby treated with standardized treatment for 24 h to ensure adequate tissue perfusion. Although there was no significant difference in norepinephrine dose between the two groups at any time, the decline trend of norepinephrine dose in group E was better. This correlates with the fact that Esmolol itself has an anti-inflammatory effect, as well as decreased myocardial oxygen consumption and improved systemic oxygen delivery after a decrease in heart rate.

A large international randomized controlled trial study showed that the use of β-blockers in the perioperative period, especially when combined with hypotension, could significantly increase the risk of death [47]. A meta-analysis aimed at recommending optimal treatment for patients with combined sepsis and heart failure concluded that the routine use of β blockers is not recommended due to the negative inotropic effect of β-blockers and lack of high-quality RCTs[48]. The present study showed that the 28-day and 90-day mortality of patients in Group E was lower than that of those in Group C, and the difference was statistically significant. Similarly, logistic regression analysis also showed that the use of Esmolol reduced the risk of death, indicating that Esmolol was beneficial for SIC patients. The study by Andrea Morelli et al. proved that the application of β-blockers to control tachyarrhythmia in patients with Septic Shock could reduce short-term mortality without increasing adverse complications [13]. In a multi-center, prospective, and randomized controlled study, it was also found that short-acting β-blockers used in patients with Sepsis could not only quickly stabilize HR, but would also not increase the incidence of adverse events or reduce mortality [49]. By analyzing the reasons, the present study found that the oxygen delivery of patients was improved after the use of Esmolol, and there was a significant correlation between oxygen delivery and patient outcomes. The reduction of mortality of patients with Esmolol was considered related to the improvement of oxygen delivery. Emanuel P. Rivers et al. affirmed that maintenance of tissue normoxia, reversal of tissue hypoxia and avoidance of tissue dysoxia mattered considerably in preventing oxygen debt, cell injury, organ failure and death [50]. The meta-analysis also found that lactic acid production during shock was largely attributed to tissue hypoxia and anaerobic glycolysis due to inadequate oxygen delivery [51]. In addition, ScvO2 improved with Esmolol, while persistently low ScvO2 reflected underlying cardiac dysfunction and was associated with increased 90-day mortality [52]. Meanwhile, Esmolol has other various potential therapeutic effects on patients with Sepsis, including improving heart function, inhibiting the production of inflammatory factors, correcting hypermetabolism, and improving coagulation disorders [53], and may therefore reduce mortality in SIC patients through a variety of action mechanisms.

It was found in a prospective cohort study that the use of Esmolol in patients with severe Sepsis could reduce the duration of mechanical ventilation [54]. C. Fuchs et al. found while studying the correlation between continuous use of β-blockers in the acute phase of severe Sepsis and Septic Shock and the 90-day mortality rate that β-blockers could reduce the length of ICU stay in patients with Sepsis Shock [45]. Compared with Group C patients, no statistically significant differences were hereby found in Group E patients in terms of mechanical ventilation time, ICU length of stay, and total length of stay. Given that pneumonia occupied a large proportion of all the causes of Sepsis in this study, it might have caused the difference in the study results.

## Strengths and limitations

This study has the following strengths: First of all, STE, which is less affected by cardiac preload and afterload and can be used to assess myocardial contractility more accurately, was used to assess myocardial contractility before and after Esmolol treatment in SIC patients for the first time; Secondly, a prospective randomized study design was adopted, which can well prevent selectivity bias and more accurately evaluate the effect of Esmolol; Thirdly, hemodynamic changes during medication were monitored in real time to ensure patient safety; Finally, PICCO parameters were firstly used to evaluate the effects of Esmolol on cardiac function. However, this study is still subject to the following limitations: Firstly, there is no definite threshold of STE for the evaluation of left ventricular dysfunction. However, the threshold value of left ventricular dysfunction in this study was obtained from previous large-scale studies, which reduced the possibility of exaggerating or covering up the fact of cardiac function; Secondly, STE was only used in this study for the continuous evaluation of changes in cardiac function of the enrolled patients for 7 days, failing to evaluate the influence of Esmolol on long-term cardiac function and hemodynamics. However, based on the use of vasoactive drugs and changes in hemodynamic parameters for 7 consecutive days, it can be reasonably proposed that long-term application of Esmolol will not cause hemodynamic instability; Finally, considering the effect of Esmolol on HR, the researchers could not be blinded in this study, but the selected parameters were all objective parameters less affected by subjective factors, and could affectively prevent information bias.

## Conclusion

In summary, the myocardial contractility did not deteriorate, and the systemic vascular tension was improved after Esmolol reducing the HR of SIC patients. The application of Esmolol in SIC patients did reduce their short-term mortality, thought it failed to reduce the mechanical ventilation time, ICU hospital stay or total hospital stay. STE does not have a threshold for SIC diagnosis, and further large-scale studies are needed for confirmation.

## Supplementary Information

**Additional file 1:** **eFigure 1.** Enrollment Flowchart, **eFigure 2.** Safety Check, **eTable 1.** Comparison between Group E and Group C at 72h , 48h and 7d in ICU.

## Data Availability

The datasets generated during and/or analyzed during the current study are available from the corresponding author on reasonable request.

## Abbreviations

SIC: Sepsis induced cardiomyopathy
STE: Speck-tracking echocardiography
PICCO: Pulse indicating continuous cardiac output monitoring
GLS: Global longitudinal strain
LVEF: Left ventricular ejection fraction
GEF: Global ejection fraction
dP/dtmx: Left ventricular systolic force index
TTE: Transthoracic echocardiography
HR: Heart rate
SBp: Systolic pressure
DBp: Diastolic pressure
MBp: Mean arterial pressure
CI: Cardiac Index
CVP: Central venous pressure
GEDVI: Global end-diastolic volume Index
ELWI: Extravascular lung water index
Lac: Lactic acid
ScVO2: Central venous oxygen saturation
Pv-aCO2: Central venous-arterial carbon dioxide difference
NT-proBNP: Amino-terminal brain natriuretic peptide
CK: Creatine kinase
CK-MB: Creatine kinase isoenzyme
TNI: Troponin I
DO2: Oxygen delivery
SOFA: Sequential organ failure assessment
Apache II: Acute physiology and chronic health
NE: Norepinephrine
SV: Stroke volume
ScvO2: Central venous oxygen saturation
