# Supplementary material for: Evaluating the effects of Esmolol on cardiac function in patients with Septic cardiomyopathy by Speck-tracking echocardiography—a randomized controlled trial
Source: BMC Anesthesiol. 2023 Feb 10;23:51. doi: 10.1186/s12871-023-01983-8 (PMC9912519; doi:10.1186/s12871-023-01983-8)
Supplement: Supplementary file 1 — Additional file 1: eFigure 1. Enrollment Flowchart, eFigure 2. Safety Check, eTable 1. Comparison between Group E and Group C at 72h , 48h and 7d in ICU. [file 12871_2023_1983_MOESM1_ESM.docx]

Additional file 1

**eFigure 1 Enrollment Flowchart**

Exclude

Surviving Sepsis campaign-2018

Age≥18years old

Sepsis-3

Diagnosis of Sepsis/Septic Shock within 1h

24h treatment target:

1. CVP>10mmHg；

2. Negative volumetric response assessment, including PLR and/or fluid challenge.

HR＜100bpm

HR≥100bpm

LVEF≤45% or GLS≥-19%

Speckle-tracking echocardiography

**Subjects：Patients with Sepsis/Septic Shock with tachyarrhythmia.**

**eFigure 2 Safety Check**

Titration interruption

**Stop events**

HR≥100bpm,MBP≥65 mmHg，LVEF＞25%

NO

Exclude

YES

Aggressive resuscitation

YES

YES

Esmolol titration

Target HR

**Stop Events**

1. Heart rate lowered 60 bpm or less, or the occurrence of Grade II or Grade III heart block;
2. Dosage of norepinephrine infusion increased by 10% within 30min;
3. Aggravation of Septic Shock after Esmolol administration reported by clinicians.

**Randomization**

In this study, a random number table method was used to perform simple randomization. Scientific research staff generated the random allocation sequence, and clinicians enrolled participants and then assigned them to interventions.

1. Draw up the serial number of 100 research objects in advance;

2. Random numbers are generated using the random number table method；

3.Subjects with odd random numbers were assigned to group E and even numbers to group C;

4. Esmolol was prescribed for group E and routine treatment for group C;

5. Keep the files for the randomization.

**Formulas of DO_2_ and VO_2_**

1. Oxygen delivery (DO_2_) is the total amount of oxygen delivered to the tissues in the entire body per minute.

DO_2_ = CaO_2_×CI×10

CaO_2_ = Hb×1.34×SaO_2_ +0.003×PO_2_

CaO_2_: arterial oxygen content; CI: cardiac index; Hb: hemoglobin; SaO_2_: arterial oxygen saturation; PO_2_: arterial partial pressure of oxygen.

2. Oxygen consumption (VO2) is the amount of oxygen extracted by the peripheral tissues per minute, representing the use of oxygen throughout the body.

VO_2_ = (CaO_2_-CvO_2_)×CI×10

CvO_2_ = Hb×1.34×SvO_2_ +0.003×PvO_2_

CvO_2_: central venous oxygen content; SvO_2_: central venous oxygen saturation; PvO_2_: central venous oxygen partial pressure.

**eTable 1 Comparison between Group E and Group C at 72h , 48h and 7d in ICU**

| **Variables** | **ICU72h** | | P | **ICU96h** | | P | **ICU7d** | | P |
| --- | --- | --- | --- | --- | --- | --- | --- | --- | --- |
|  | **Group E** | **Group C** |  | **Group E** | **Group C** |  | **Group E** | **Group C** |  |
| **NE**  **(μg•kg^-1^•min^-1^)** | 0.67±0.74 | 0.96±0.89 | 0.069 | 0.65±0.91 | 0.88±1.02 | 0.234 | 0.065±0.12 | 0.033±0.091 | 0.213 |
| **HR(bpm)** | 86.04±6.99 | 99.90±14.89 | 0.000^*^ | 85.56±6.94 | 98.28±16.21 | 0.000^*^ | 83.55±7.71 | 85.74±7.20 | 0.293 |
| **MBP(mmHg)** | 84.50±11.17 | 85.98±9.71 | 0.481 | 84.62±10.13 | 83.61±10.82 | 0.985 | 89.17±11.68 | 86.47±9.24 | 0.274 |
| **GLS** | -12.59±3.55 | -10.71±3.83 | 0.012^*^ | -13.13±3.79 | -12.54±4.02 | 0.452 | -17.09±6.66 | -16.02±7.14 | 0.440 |
| **E/e’** | 13.79±4.48 | 13.78±4.28 | 0.993 | 13.69±4.47 | 14.46±3.67 | 0.355 | 12.38±5.73 | 12.57±6.31 | 0.875 |
| **LVEF(%)** | 47.99±12.45 | 47.28±11.73 | 0.766 | 47.10±12.05 | 49.25±10.20 | 0.338 | 54.68±12.62 | 53.90±12.70 | 0.759 |
| **CI(L/min/m^2^)** | 3.35±0.97 | 3.27±0.84 | 0.652 | 3.48±1.18 | 3.37±0.97 | 0.625 | 3.39±1.12 | 3.42±0.86 | 0.898 |
| **SVRI**  **(dyn•s•cm^-5^•m^-2^)** | 2646.4±733.2 | 2479.0±606.9 | 0.217 | 2412.2±601.6 | 2384.1±558.1 | 0.809 | 2271.2±679.1 | 2311.5±613.3 | 0.788 |
| **GEDI(ml/m2)** | 747.48±62.99 | 746.90±73.45 | 0.966 | 747.28±70.08 | 742.02±73.84 | 0.716 | 757.64±87.36 | 760.62±76.16 | 0.875 |
| **EWLI(ml/kg)** | 9.10±2.39 | 9.32±3.78 | 0.729 | 8.48±3.20 | 8.78±4.11 | 0.685 | 9.56±3.44 | 9.38±3.14 | 0.823 |
| **GEF(%)** | 27.44±5.12 | 25.28±5.25 | 0.040^*^ | 27.68±5.53 | 26.04±5.54 | 0.135 | 25.83±5.85 | 27.33±5.75 | 0.266 |
| **dP/dtmx**  **(mmHg/s)** | 1368.0±292.1 | 1242.6±328.5 | 0.047^*^ | 1379.9±299.5 | 1309.8+315.6 | 0.292 | 1211.1±358.8 | 1247.9±360.1 | 0.659 |
| **CVP(mmHg)** | 10.72±2.63 | 9.90±2.86 | 0.139 | 10.10±2.84 | 9.48±2.70 | 0.266 | 10.50±3.99 | 9.28±2.78 | 0.127 |
| **Lac(mmol/L)** | 2.76±1.47 | 2.63±1.44 | 0.664 | 2.38±1.58 | 2.28±1.57 | 0.764 | 1.82±1.09 | 1.89±0.96 | 0.768 |
| **VO2(ml/min)** | 245.42±80.27 | 251.96±77.43 | 0.679 | 240.58±68.00 | 252.46±78.48 | 0.421 | 219.47±55.88 | 213.31±65.59 | 0.664 |
| **DO2(ml/min)** | 649.02±157.36 | 610.40±127.66 | 0.181 | 655.36±131.86 | 612.06±122.44 | 0.092 | 713.56±87.56 | 692.67±87.44 | 0.305 |
| **P(v-a)CO2**  **(mmHg)** | 4.34±1.76 | 3.77±1.82 | 0.112 | 3.44±1.52 | 3.73±1.37 | 0.326 | 3.21±1.68 | 3.01±2.13 | 0.653 |

NE: norepinephrine; HR: heart rate; bpm: beats per minute; MBP: mean blood pressure; GLS: left ventricular global longitudinal systolic strain; LVEF: left ventricular ejection fraction; CI: cardiac index; SVRI: systemic vascular resistance index; GEDI: global end diastolic volume index; GEF: global ejection fraction; dP/dtmx: left ventricular contractility index; CVP: central venous pressure; VO2: oxygen consumption; DO2: oxygen delivery; P(v-a)CO2: central venous-arterial carbon dioxide partial pressure difference; *: p<0.05.

At 72h in ICU, parameters including GLS, GEF and dP/dtmx reflecting myocardial contraction for patients in Group E were better than those in Group C [(-12.59±3.55) VS (-10.71±3.83), (27.26±4.82) VS (25.28±5.25), (1368.0±292.1) VS (1242.6±328.5), p<0.05]. At 96h and 7d in ICU, there was no statistical difference in parameters between the two groups (p>0.05).
